# Platelets as Mediators of Neuroinflammation and Thrombosis

**DOI:** 10.3389/fimmu.2020.548631

**Published:** 2020-10-06

**Authors:** Elias Rawish, Henry Nording, Thomas Münte, Harald F. Langer

**Affiliations:** ^1^ University Hospital Schleswig-Holstein, Medical Clinic II, University Heart Center Lübeck, Lübeck, Germany; ^2^ DZHK (German Centre for Cardiovascular Research), partner site Hamburg/Kiel/Lübeck, Lübeck, Germany; ^3^ University Hospital Schleswig-Holstein, Clinic for Neurology, Lübeck, Germany

**Keywords:** platelets, neuroinflammation, thrombosis, stroke, therapy, cytokines, encephalomyelitis, Alzheimer's

## Abstract

Beyond platelets function in hemostasis, there is emerging evidence to suggest that platelets contribute crucially to inflammation and immune responses. Therefore, considering the detrimental role of inflammatory conditions in severe neurological disorders such as multiple sclerosis or stroke, this review outlines platelets involvement in neuroinflammation. For this, distinct mechanisms of platelet-mediated thrombosis and inflammation are portrayed, focusing on the interaction of platelet receptors with other immune cells as well as brain endothelial cells. Furthermore, we draw attention to the intimate interplay between platelets and the complement system as well as between platelets and plasmatic coagulation factors in the course of neuroinflammation. Following the thorough exposition of preclinical approaches which aim at ameliorating disease severity after inducing experimental autoimmune encephalomyelitis (a counterpart of multiple sclerosis in mice) or brain ischemia-reperfusion injury, the clinical relevance of platelet-mediated neuroinflammation is addressed. Thus, current as well as future propitious translational and clinical strategies for the treatment of neuro-inflammatory diseases by affecting platelet function are illustrated, emphasizing that targeting platelet-mediated neuroinflammation could become an efficient adjunct therapy to mitigate disease severity of multiple sclerosis or stroke associated brain injury.

## Introduction

Platelets, also called thrombocytes, are produced by megakaryocytes as tiny anucleate cells that, however, contain mRNA and a translational machinery; hence, they are capable of synthesizing proteins (1). After leaving the bone marrow, platelets circulate for about 7 to 10 days (2), subsequently they are eliminated by macrophages mainly in the spleen and liver (3). Platelets are classically regarded as the major actor of primary hemostasis. Thus, their main function is stopping hemorrhage following vascular injury by rapidly binding to damaged blood vessels and forming thrombi (4). However, activated platelets also aggregate during atherosclerotic plaque erosion or rupture, stimulating thrombus formation and promoting severe atherothrombotic diseases such as acute limb ischemia or myocardial infarction (5, 6).

Beyond their importance in hemostasis and thrombosis, an increasing body of evidence points to a crucial role of platelets for inflammatory and immune responses (7, 8). For instance, platelets have been demonstrated to mediate inflammatory response in arthritis (9) or sepsis (10). Furthermore, thrombosis itself is pathophysiologically linked with inflammation in most diseases associated with ischemia-driven organ damage (11, 12). Accordingly, platelets have been shown to be of decisive importance for thrombo-inflammatory diseases such as stroke (13). Emerging evidence indicates a detrimental role of platelets not only in the context of neurovascular thrombosis but also in other neuro-inflammatory conditions, e.g., multiple sclerosis (MS) (14). Considering the severity of these diseases and the diminished patients’ quality of life, there is an urgent need for novel therapeutic options.

Therefore, this review summarizes recent insights into the pathophysiological role of platelet receptors and related downstream signaling as well as platelet-mediated cell-cell interactions in neurovascular inflammation. Furthermore, translational and clinical applications are portrayed in order to delineate future therapeutic strategies for neuro-inflammatory diseases such as stroke or MS by targeting platelet function.

## Mechanisms of Platelet-Mediated Thrombosis and Inflammation

As injuries require both an efficient hemostasis and an inflammatory immune response against entering pathogens, the close linkage between inflammatory and thrombotic processes is assumed to have an evolutionary origin (15, 16). Following vasoconstriction, platelets are the first immunomodulatory cells at the side of injury sealing damaged blood vessels by aggregation and forming a thrombus. Thereby, platelets promote inflammatory activity by an intimate crosstalk with leukocytes (17): In case of vascular injury, neutrophils or monocytes are suggested to interact either with endothelium-adherent platelets or, prior to endothelial contact, directly with platelets forming platelet-leukocyte-aggregates (PLA) which are recruited to the inflamed vessel wall (18). Thus, platelets orchestrate the inflammatory response by regulating the further adhesion of innate immune cells to the inflamed endothelium, which is regarded to be critical for the atherosclerotic disease process (19). For instance, macrophage pro-inflammatory cytokine secretion is enhanced following interaction with activated platelets *in vitro*, suggesting that the presence of activated platelets at sites of inflammation exacerbates pro-inflammatory macrophage activation (20). Further molecular mechanisms and receptors participating in the crosstalk between innate immune cells and platelets are outlined below.

### Interaction of Platelets With Cells of Acquired Immunity

In addition to the interaction with the innate immune system, a crosstalk between platelets and B cells as well as T cells has been reported (21). Platelets have been demonstrated to induce B cell isotype switching (22). When platelets are co-incubated with B-cells *in vitro*, B-cells increase their production of IgG1, IgG2, and IgG3, indicating that platelet content can contribute to B-cell function and alter adaptive immunity (23). T-cell activation increases platelet aggregation *via* both T cytolytic and T helper cells mediated by platelet GPIIb/IIIa, CD40L, and lymphocyte integrin alpha M (24). Experimental approaches indicate that platelets may facilitate the recruitment of lymphocytes to an injured vessel at a site of vascular inflammation, constituting a central step in T-cell trafficking (25). Furthermore, activated platelets can modulate T-cell functions by releasing platelet factor 4 (PF4, chemokine [C-X-C motif] ligand 4, CXCL4), RANTES (CC-chemokine ligand 5, CCL5), or serotonin (26–28). For instance, PF4 is necessary for the limitation of Th17 expansion and differentiation (29). Serotonin, which is largely stored in platelet δ-granules, can activate naïve T-cells to stimulate their proliferation (26, 27). Hence, the interaction between platelets and lymphocytes should be considered as a relevant intersection in thrombo-inflammatory processes; therefore, receptors in platelet-immune cell interaction are further delineated in the following chapters.

### Platelets and the Humoral Immunity

Platelets have been identified as a major source of chemokines and cytokines at the site of inflammation (30). For instance, activated platelets mediate inflammatory signaling and cell recruitment by secreting RANTES, PF4, and IL-1β (31, 32). Emphasizing the role of platelets at the intersection between thrombosis and inflammation, their IL-1 activity yielded an exacerbation of atherosclerotic lesions as well as an upregulation of adhesion molecules and chemokine expression by human umbilical vein endothelial cells (HUVECs) (33, 34). Remarkably, platelet activation of brain endothelium *via* IL-1 has been recognized to promote the release of CXCL1, which plays an essential role in the subsequent leukocyte recruitment during neuroinflammation (35, 36). Furthermore, platelets contain nuclear factor kappa-light-chain-enhancer of activated B cells (NF-κB) family members (37) that are critically involved in both inflammatory and thrombotic responses, which has recently been reviewed elsewhere (38). Moreover, a crosstalk between platelets and the complement system conduces to platelet-mediated inflammation (39, 40). Thus, the interaction of platelets with the complement system will be discussed here in the context of neurovascular inflammation, whereas further aspects have been comprehensively portrayed elsewhere (11).

Overall, a broad range of mechanisms contribute to platelet-mediated inflammation, revealing several fields for future research on diseases associated with thromboinflammation.

## Platelet Receptors and Interactions in the Context of Thromboinflammation

Both mechanisms of hemostasis respectively thrombosis and mechanisms of platelet-mediated inflammation require a close interaction of platelets with endothelial and immune cells but also with the extracellular matrix. Platelet adhesion receptors constitute the major determinants of these interactions. Commonly, four types of platelet receptors are considered as being crucial for hemostasis, thrombosis and inflammation: integrins, leucine-rich glycoproteins (GPs), selectins as well as receptors of the immunoglobulin type.

Under flow conditions, especially at high shear stress (>500 s^−1^) as in small arteries and arterioles, the initial adhesion of platelets to the injured blood vessel wall requires the interaction between immobilized von Willebrand factor (vWF) on the surface of the endothelium or in the subendothelial matrix with its platelet receptor GPIbα, which is part of the GPIb-IX-V complex (41, 42). In addition, exposed subendothelial collagen binds reversibly to platelet GPIa/IIa recptor (also known as integrin α_2_β_1_) and GPVI receptor, a member of the immunoglobulin superfamily (43). The firm binding of collagen to platelet GPVI receptor allows resistance towards high shear rates, and furthermore, induces platelet activation by a rise in the cytosolic Ca^2+^ concentration. Thus, platelet shape changes and P-selectin, platelet endothelial adhesion molecule-1 (PECAM-1), vWF, and fibrinogen from α-granules as well as ADP, calcium and serotonin from dense granules are released, which in turn fuels further platelet activation *via* autocrine and paracrine signaling by G-protein coupled receptors (44, 45). The final common pathway of platelet activation is the conformational change in platelet GPIIb/IIIa (also named integrin α_IIb_β_3_) receptor which results in the cross-link of fibrinogen or vWF between GPIIb/IIIa receptors, leading to platelet aggregation (46). Thereby, platelet integrin receptors α_2_β_1_, α_5_β_1_, and α_6_β_1_ stabilize thrombus formation by binding to components of the extracellular matrix (47–49).

Importantly, platelet-mediated leukocyte recruitment is initiated by binding of platelet P-selectin to leukocyte P-selectin glycoprotein ligand-1 (PSGL-1) (50), inducing activation of β_1_ and β_2_ integrins and increasing adhesion of leukocytes to activated endothelium (51). Contrariwise, PSGL-1 on platelets can interact with P-selectin on endothelial cells as well (52). Interestingly, fractalkine (CX3CL1) expressed by inflamed endothelial cells can bind to the fractalkine receptor CX3CR1 on platelets triggering an increased P-selectin expression on platelets, thereby initiating local accumulation of leukocytes (53). Besides, another member of platelet selectin family, C-type lectin-like receptor-2 (CLEC-2), is thought to be a major player in thrombo-inflammatory disorders (54): Using a murine model of systemic *Salmonella Typhimurium* infection, it has been demonstrated that inflammation in several tissues triggers thrombosis within vessels *via* activation of CLEC-2 on platelets by its ligand podoplanin exposed to the vasculature following breaching of the vessel wall (55). Thus, targeting CLEC-2 could be a potential therapeutic approach in order to control infection-driven thrombosis. Interestingly, mice with general inducible deletion of CLEC-2 or platelet-specific deficiency in CLEC-2 were protected against deep vein thrombosis (56). With respect to neuroinflammation, it has recently been demonstrated that inhibition of spleen tyrosine kinase (Syk), which is part of the CLEC-2 downstream pathway, reduces neuroinflammation and infarct volume after ischemic stroke in mice (57). On the other hand, platelet CLEC‐2 has been shown to diminish trauma‐induced neuroinflammation and restore blood–brain barrier integrity following controlled cortical impact injury (58). Thus, the potential of CLEC-2 as a target in the context of neuroinflammation remains uncertain.

GPIb interacts with the leukocyte integrin macrophage-1 antigen (Mac-1, also known as α_M_β_2_ or CD11b/CD18); thereby promoting a firm leukocyte/platelet adhesion (59). Accordingly, GPIb inactivation leads to reduced leukocyte adhesion to the vessel wall as well as to diminished development of atherosclerotic lesions in atherosclerosis-prone apolipoprotein E-deficient (ApoE^−/−^) mice (60). Underlining the importance of GPIb for cerebral inflammation, it has recently been reported that platelet-mediated neutrophil infiltration to the brain can be reduced by 44% when platelet receptor GPIb is blocked in an *in vivo* model of lipopolysaccharide (LPS)-induced neuroinflammation (61).

In addition to GPIb, fibrinogen bound to platelet GPIIb/IIIa receptor can also interact with leukocyte Mac-1 in a platelet activating factor (PAF) regulated manner (62). Mac-1 on monocytes and neutrophils were identified as critical molecular links between inflammation and thrombosis, e.g., in myocardial infarction (62) or else in thrombotic glomerular injury (63). Strikingly, recent experimental approaches have demonstrated that both Mac-1 deficiency and mutation of the Mac-1-binding site for GPIb delay thrombosis after carotid artery injury without affecting parameters of hemostasis (64). Thus, targeting Mac-1-mediated leukocyte/platelet interaction is suggested to have an anti-thrombotic therapeutic potential with reduced bleeding risk (64).

Fascinatingly, platelet-derived microparticles (PMPs), that are generated from the plasma membrane upon platelet activation, harbor functional GPIIb/IIIa receptors which can be acquired by neutrophils and cooperate in neutrophil-induced inflammation *via* NF-κB activation (65). Accordingly, GPIIb/IIIa receptor antagonists reduced thrombo-inflammatory processes, as the formation of PLA, in patients with acute coronary syndromes undergoing percutaneous coronary intervention (PCI) (66). In the course of neurovascular inflammation, magnetic resonance imaging (MRI) studies demonstrated the presence of activated platelet GPIIb/IIIa receptor in the inflamed brain of malaria-infected mice using a specific antibody conjugated to iron oxide microparticles (67). Elevated PMP levels have also been detected in stroke patients (68, 69). However, a prognostic value of plasma PMP on recurrence of stroke, neurological outcome or survival is not established (70). Therefore, the pathophysiological significance of PMPs in stroke remains elusive.

In addition, a contribution of platelet GPVI receptor to thrombo-inflammatory disorders has been repeatedly shown (54). For instance, inhibition of GPVI causes a reduction in inflammatory cell recruitment and infarct size following myocardial ischemia-reperfusion injury by improving microperfusion (71). Further receptors of the immunoglobulin superfamily are also of importance for platelet interactions: Under low shear stress platelets interact with leukocytes by binding of intercellular adhesion molecule 2 (ICAM-2, also known as CD54) on platelets to lymphocyte function-associated antigen 1 (LFA-1) on leukocytes (72). Moreover, junctional adhesion molecules-C (JAM-C) expressed on platelets are critical for the recruitment of DCs to the vascular wall *via* an interaction with Mac-1 on DCs (73).

Intriguingly, platelets express functional toll-like receptors (TLRs) (74), which are a major family of receptors that recognize pathogen-associated molecular patterns (PAMPs). In the context of thrombosis and inflammation, it has lately been revealed that platelet TLR2 can accelerate thrombosis in hyperlipidemic ApoE^−/−^ mice (75). Further interactions of platelet TLRs in thrombo-inflammatory responses have been extensively reviewed elsewhere (76). In addition, complement receptors for C3a (C3aR) and C5a (C5aR) have been detected on platelets (77, 78); whereby platelet C5aR has been correlated to markers of platelet activation (79). Interestingly, a strong positive correlation of platelet C3aR expression with activated GPIIb/IIIa has been reported in thrombi obtained from patients with myocardial infarction (77). Besides, C3 on platelets has been shown to be elevated in ischemic stroke (80), further indicating an intimate relation between the complement system and platelets in cardiovascular diseases.

CD40 and CD40L (a member of the tumor-necrosis factor [TNF] superfamily, also named as CD154) are a receptor and its corresponding ligand which are decisive mediators of interactions between lymphocytes and antigen-presenting cells (81). Remarkably, CD40L has been implicated in numerous inflammatory conditions, such as atherothrombotic diseases (82) or else neuro-inflammatory disorders including cerebral malaria (83), Alzheimer’s disease (AD) (84, 85) as well as HIV-associated CNS-inflammation (86). CD40L is present in the granules of resting platelets (87) and is rapidly translocated to the platelet surface upon activation (88). Platelet-expressed CD40L has been indicated to affect DCs, B cells as well as T cells, providing a crosslink between innate and adaptive immunity (89). Moreover, platelet CD40L interacts with CD40 on endothelial cells, promoting secretion of chemokines, such as IL-8 and monocyte chemotactic protein 1 (MCP1) as well as expression of adhesion molecules such as E-selectin, vascular cell adhesion molecule 1 (VCAM-1), and ICAM-1 (88). Platelet CD40L also contributes to neuroinflammation by inducing activation of astrocytes and microglia (90). Furthermore, activated platelets express soluble CD40L (sCD40L) which in turn induces endothelial secretion of IL-6 and surface expression of P-selectin. Thus, CD40L-mediated interactions promote migration of leukocytes to the site of vascular injury and subsequent adhesion (46).

The potential role of platelets in (neuro-) inflammation can be underlined by findings from neurologic complications of malaria. In Patients with Malaria, platelets were observed binding directly with and killing intraerythrocytic parasites of each of the *Plasmodium* species studied, a process which seems to be dependent on PF4 (91). In fact, thrombocytopenia is a hallmark of blood-stage plasmodium infection, and malaria is characterized by a procoagulant state that is most pronounced in Plasmodium falciparum (Pf) infections, the most virulent of the 5 species of Plasmodium infecting humans (92, 93). Other studies do not favor the hypothesis of direct killing of bacteria by platelets, but rather suggest an indirect inflammation-activating effect. Recently, it was demonstrated that platelets elicit the pathogenesis of malaria and that platelet CD40 is a key molecule in this process using an adoptive transfer model of WT platelets into CD40-KO mice, which are resistant to experimental cerebral malaria, whereby experimental cerebral malaria mortality and symptoms in CD40-KO recipients was partially restored (94). Platelet depletion experiments demonstrated that platelets contribute to the inflammatory response of experimental cerebral malaria, particularly in the early phase (95, 96).

Summarized, the diversity of platelet receptors participating in platelet interactions reveals various interesting targets within the context of platelet-mediated inflammation. Thus, the most promising targets during neurovascular inflammation are illuminated below.

### Platelets, the Coagulation Cascade and Thrombosis

The classical plasmatic coagulation cascade of secondary hemostasis consists of the contact activation (intrinsic) pathway, the tissue factor (TF; extrinsic) pathway as wells as the final common pathway (97). This traditional theory of blood coagulation is suitable for describing coagulation *in vitro* but it has flaws as a model of the hemostatic process *in vivo* (98). For instance, the model cannot explain why hemophilia A patients bleed although they have an intact “extrinsic” pathway (99). Thus, a current cell biological model of coagulation divides coagulation into three overlapping phases: Firstly, the initiation phase, in which low amounts of active coagulant factors are generated. At this stage, TF in damaged vessel binds “extrinsic” factor (F)VIIa to activate “intrinsic” FIX as well as FX. In the second stage, the amplification phase, levels of active coagulation factors, such as thrombin are boosted, leading to platelet-activation by cleaving protease-activated receptor 1 (PAR1). Finally, in the propagation phase, coagulation factors bind to procoagulant membranes of activated platelets driving formation of fibrin clots (100). Hence, according to the cellular model of coagulation, the “intrinsic” pathway mainly serves as an amplification loop initiated by the TF pathway (100).

Nevertheless, one should not undervalue the role of the “intrinsic” pathway. For instance, platelets are able to activate FXII as they contain negatively charged polyphosphates (polyP) which can be externalized onto the cell membrane upon platelet activation (101). Thereby, platelets promote subsequent activation of plasma kallikrein, FIX and further downstream coagulation factors of the ‘intrinsic’ pathway (102). Interestingly, polyP-dependent FXII activation does not yield a faster clot formation, but rather an increased fibrin clot stability (100). Accordingly, high levels of FXII were associated with thrombosis, whereas FXII inhibition reduces thrombus formation in mice (103) as wells as in primate thrombosis model (104). However, FXII deficiency is not associated with bleeding (105). Thus, targeting FXII might be a pharmacological option in order to reduce arterial thrombosis risk without influencing hemostasis (106). In line with this, deficiency or inhibition of FXII protected mice from ischemic brain injury (107, 108): Using a transient middle cerebral artery occlusion (tMCAO) modell (109), Kleinschnitz et al. found that the volume of infarcted brain in FXII-deficient (FXII^−/−^) and FXII inhibitor–treated mice are substantially less than in wild-type (WT) controls, without an increase in infarct-associated hemorrhage (107). Furthermore, treating FXII^−/−^ mice with human FXII “rescued” the WT phenotype regarding infarct volume as well as intravascular fibrin and platelet deposits leading to vessel occlusion (107). The importance of FXII in neurovascular thrombo-inflammatory diseases is underlined by the notion that a lack of its downstream coagulation factor XI has protective effects against stroke in humans (110) as well as in tMCAO mice model (107). Besides, activation of the kallikrein–kinin system (KKS) by FXII stimulates the production of the potent proinflammatory peptide bradykinin (111). Strikingly, bradykinin receptor B1 knockout mice have been shown to develop reduced brain infarct volumes after tMCAO compared with WT controls (112); thereby, crosslinking FXII-mediated thrombotic activity to inflammation.

Further strengthening the hypothesis that an interaction of platelets with the intrinsic pathway of coagulation could contribute to neurovascular inflammation and stroke, Choi et al. have demonstrated that polyP secreted by activated human platelets also accelerates factor XI activation mediated by thrombin (113). However, a potential direct crosslink between synthesis of polyP in platelets and the involvement of the coagulation cascade in stroke has not yet been investigated, as the protein(s) responsible for the polyP synthesis in higher eukaryotic species have not been identified so far (114). Nevertheless, neutralizing polyP using recombinant *Escherichia coli* exopolyphosphatase (PPX) (115) in tMCAO mice model could be an absorbing alternative experimental approach.

Beside interacting with the “intrinsic” pathway of the coagulation system, activated platelets may release TF after *de novo* synthesis (116). However, this assumption is the subject of a controversial discussion, as other, flow cytometric based, investigations indicated that no TF would be expressed on activated platelets (117). Only recently has the debate whether platelets can release TF by themselves been portrayed elsewhere in detail (118, 119). Regardless of this debate, platelet CD40L expression has been reported to induce monocyte expression of tissue factor, which in turn activates the extrinsic coagulation cascade (120); thus, emphasizing the intimate interaction between platelets, immune cells and the plasmatic coagulation system.

## Contribution of Platelets to Neurovascular Thrombosis and Thromboinflammation

Stroke is the second leading cause of death and third most common cause of disability worldwide. Approximately 80% of all strokes are caused by cerebral ischemia, whereas hemorrhagic events account for the remainder (121). Most non­lacunar ischemic strokes are of thromboembolic origin, with common sources of embolism being cardiac diseases, particularly atrial fibrillation, as well as symptomatic extracranial large artery atherosclerosis (122). Immediately after intracranial vessel occlusion by an embolus the lack of oxygen and glucose in the affected brain tissue leads to focal neurological deficits such as hemiparesis or aphasia. The mainstay of treatment for ischemic stroke is prompt recanalization by thrombolysis or mechanical thrombectomy (123). Unfortunately, many patients display secondary infarct growth despite successful vessel recanalization. As indicated above, reperfusion injury has been attributed to the thrombo‐inflammatory activity of platelets and immune system cells (124). In particular, evidence suggests that T cells crucially contribute to reperfusion injury in stroke as immunodeficient Rag1^−/−^ mice, which are lacking of T cells and B cells, developed smaller infarcts after tMCAO compared with WT mice (125, 126). Additionally, the critical contribution of T cells to brain injury in stroke had been further highlighted, as adoptive transfer of T cells, to Rag1^−/−^ mice restored susceptibility to reperfusion injury after tMCAO (125, 126). Later on, particularly Forkhead box P3 (FOXP3)­positive regulatory T (T_reg_) cells have been identified as the detrimental type of T cells in ischemia–reperfusion injury (127). Strikingly, the removal of platelets from the circulation of Rag1^−/−^ mice that received adoptive transfer of T_reg_ cells has led to infarcts that were as small as in naive Rag1^−/−^ mice after tMCAO (127). Thus, this investigation of Kleinschnitz et al. first discovered that the harmful effects of T cells in ischemia–reperfusion depend on platelets; thereby, underlining the determining role of platelets in stroke-associated thromboinflammation in a compelling fashion.

However, blockade of platelet GPIIb/IIIa receptor has led to intracranial hemorrhage and has not reduced cerebral infarct sizes following tMCAO in mice (128). In line with this, anti-GPIIb/IIIa treatment of patients with acute ischemic stroke is associated with a significant risk of intracranial hemorrhage with no evidence of any reduction in death or disability in survivors (129). Thus, final platelet aggregation *via* GPIIb/IIIa is not the critical mechanism underlying thromboinflammation and reperfusion injury in stroke.

In view of the delineated GPIb-mediated interaction between platelets and leukocytes, the vWF/GPIb axis could, however, be a potential pathomechanism of thromboinflammation in stroke. Indeed, blockade of vWF binding site on GPIb using p0p/B has reduced infarct size and improved reperfusion as well as neurological status after tMCAO (128). These effects were detected both in prophylactic (1 h before tMCAO) and therapeutic (1 h after tMCAO) setting. Furthermore, it has recently been revealed that inhibition of GPIb not only reduces infarct size but also limits the local inflammatory response in the ischemic brain, since levels of inflammatory cytokines and infiltration of T cells as well as macrophages were reduced after GPIb inhibition (130). Notably, GPIb blockade has not been accompanied by an increase in intracerebral bleeding complications (128). In line with these findings, both GPIb-deficient (131) and vWF-deficient mice (130) displayed smaller infarcts and a better neurological outcome than WT mice after tMCAO. Accordingly, apoptosis in the brain tissue was reduced in GPIb-deficient mice (132). Thereby, Schleicher et al. revealed that platelets induce neuronal apoptosis *via* expression of membrane bound Fas ligand (FasL) (132).

Exemplifying the suggested importance of the interaction between leukocyte Mac-1 and platelet GPIb in neurovascular thromboinflammation, mice deficient in Mac-1 have been found to be less susceptible to cerebral ischemia (133). Further supporting the role of the vWF-GPIb axis, mice lacking *A disintegrin and metalloprotease with thrombospondin type 1 repeats 13* (ADAMTS13), an enzyme that cleaves highly thrombogenic large vWF to smaller and less active vWF, are more vulnerable to brain damage following tMCAO (134). The reperfusion injury in ADAMTS13-deficient mice has further been accompanied by an increased accumulation of immune cells in the ischemic brain (134), underscoring the role of inflammation in neurovascular thrombosis. In accordance with experimental findings, high serum levels of vWF in patients as well as autoantibodies against ADAMTS13 have been identified as risk factors for stroke (135, 136).

As outlined above, further platelet activation following vWF-GPIb interaction is mainly driven by GPVI. Displaying GPVI as another key player in the neuronal damage during stroke, its inactivation by GPVI antibody (JAQ1) caused reduced brain infarct volumes after tMCAO without increasing the risk for cerebral hemorrhage (128). In addition, Kraft et al. have demonstrated that both GPVI and GPIb blockade protect from stroke in aged mice, mice with diabetes mellitus as well as hypertensive mice, suggesting that targeting GPVI or GPIb may be a future therapeutic option for patients with accompanying common metabolic diseases (137). Accordingly, inhibition of phospholipases D1 and D2, which are downstream signals of the vWF‐GPIb axis in platelets (138), has yielded reduced susceptibility to stroke progression following tMCAO again without increasing bleeding risk (139). Likewise, the blockade of GPVI dependent downstream pathways has been reported to protected from stroke progression after tMCAO by reducing Ca^2+^ responsiveness in platelets (140). Platelet granule secretion depends on intracellular Ca^2+^ mobilization (141) and has been demonstrated to be crucial in ischemic-reperfusion injury (142). For instance, mice showing deficiency in both platelet dense granule secretion (143) and α-granule secretion (144) were protected from cerebral ischemia after tMCAO without observation of intracranial hemorrhage.

A role in cerebral ischemia-reperfusion injury has also been described for CD40L. According to Ishikawa et al., both CD40 and CD40L-deficient mice showed reduced infarct volume after tMCAO compared with WT mice (145). This notion was accompanied by diminished platelet/leukocyte adhesion, blood cell recruitment and neurovascular permeability in CD40(L)-deficient mice. Supporting the role of CD40/CD40L in thromboinflammation, plasma levels of sCD40L were significantly higher in patients with acute cerebral ischemia compared with controls. Furthermore, CD40 expression on monocytes was higher in stroke group, accompanied by significantly increased amount of prothrombotic platelet-monocyte aggregates (146).

The insinuated contribution of the complement system to platelet-mediated thromboinflammation has recently been depicted in a gripping fashion: Using C3aR^-/-^ mice, Sauter et al. demonstrated not only that complement activation fragment C3a regulates bleeding time but also that C3aR^-/-^ mice are less prone to experimental stroke and myocardial infarction (77). Notably, reconstitution of C3aR^-/-^ mice with C3aR^+/+^ platelets has restored bleeding time and susceptibility to reperfusion injury after tMCAO (77). In this context, it is worthwhile to mention the association of high serum levels of complement lectin pathway activator mannan-binding lectin (MBL) with cardiovascular diseases such as stroke (147). In accordance, infarct volumes and neurological deficits after tMCAO were smaller in MBL^-/-^ mice than in WT controls. Remarkably, Orsini et al. have recently demonstrated that protection of MBL^-/-^ mice against cerebral ischemia-reperfusion injury is accompanied by a less inflammatory phenotype of platelets as indicated by reduced IL-1α content in platelets (148). Furthermore, cultured human brain endothelial cells subjected to a lack in oxygen/glucose and exposed to platelets from MBL^-/-^ mice displayed less cell death and lower CXCL1 release than those exposed to WT platelets (148). These observations distinctly underscore the importance of the pathophysiological crosstalk between platelets, brain endothelial cells, and mediators of the immune system in reperfusion injury of the brain.

**Figure 1:**
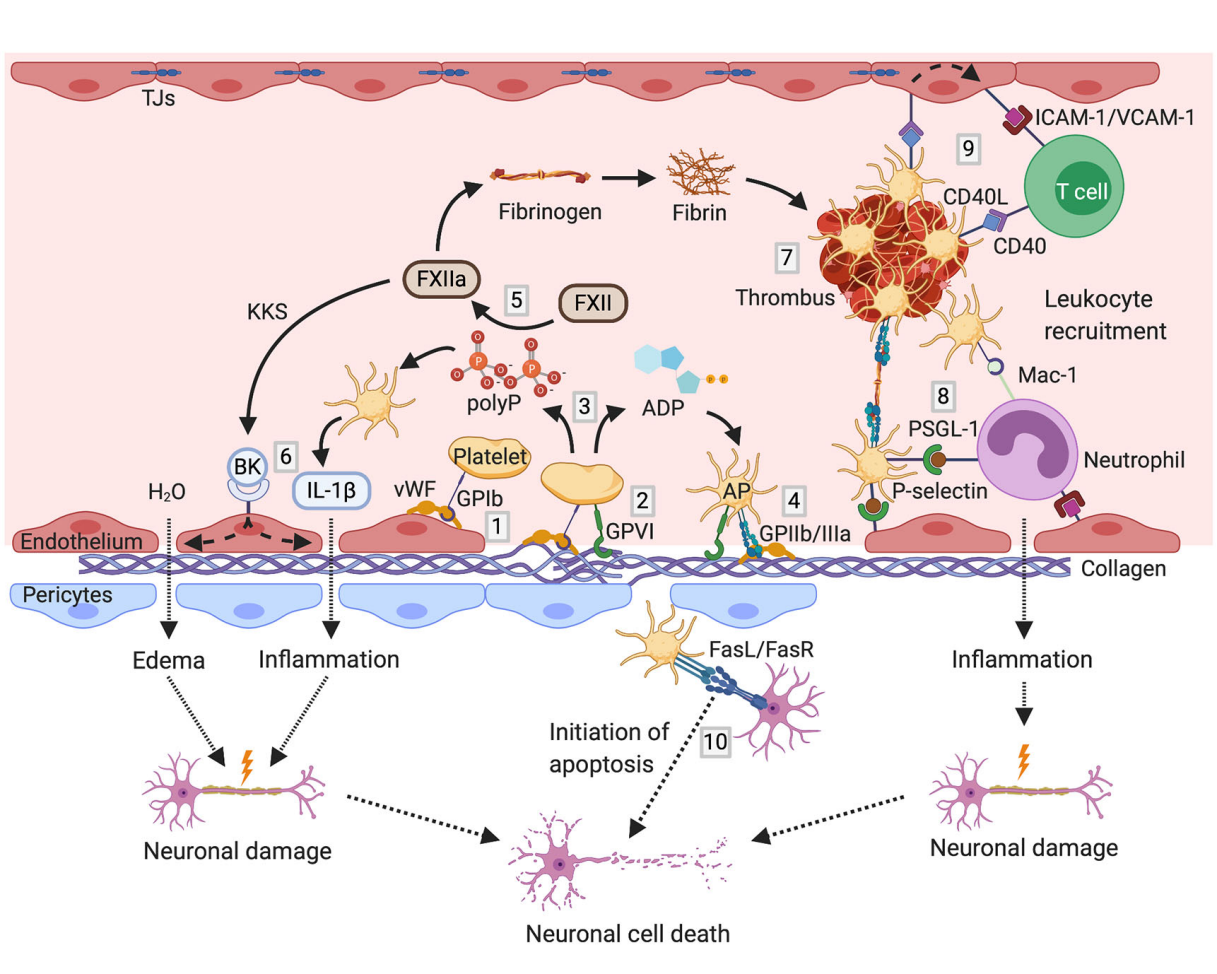
Mechanisms of thromboinflammation in stroke; partially adopted and modified from (206, 207): Initial tethering of platelets to the extracellular matrix or endothelium at the site of ischemic vascular injury is mediated by GPIb binding to exposed vWF (1). The interaction between platelet GPVI receptor and subendothelial collagen triggers platelet activation (2). Activated platelets release paracrine factors including ADP and polyP (3), promoting functional upregulation of GPIIb/IIIa (4). Negatively charged polyP activate coagulation FXII (5). FXIIa stimulates the activation of the KKS, thereby promoting the release of the proinflammatory peptide bradykinin. In company with further cytokines such as IL-1ß, bradykinin causes endothelial cell damage leading to vascular edema and neuronal damage (6). On the other hand, FXIIa initiates the intrinsic coagulation pathway, triggering thrombus formation by fibrin engenderment (7). Activated platelets mediate thromboinflammation also by recruitment of leukocytes *via* binding of platelet P-selectin to leukocyte PSGL-1 as well as *via* GPIb/Mac-1 interaction (8). Stable tethering of leukocytes to the vessel wall is achieved by the interaction between platelet CD40L with CD40 on endothelial cells, promoting the expression of adhesion molecules such as ICAM-1 and VCAM-1 on endothelial cells (9). Thereby, platelets orchestrate the infiltration of immune cells into the brain parenchyma leading to further neuronal damage. Besides, platelets can initiate apoptosis *via* the expression of death receptor FasL on their surface (10). ADP, adenosine diphosphate; AP, activated platelet; BK, bradykinin; FasL, Fas ligand; FasR, Fas receptor; FXII, factor XII; GP, glycoprotein; ICAM-1, intercellular adhesion molecule 1; IL, interleukin; KKS, kallikrein–kinin system; Mac-1, macrophage-1 antigen; polyP, polyphosphates; PSGL-1, P-selectin glycoprotein ligand-1; TJs, tight junctions; VCAM-1, vascular cell adhesion protein 1; vWF, von Willebrand factor. Figures created with BioRender.com.

Taken together, particularly GPIb, GPVI, C3aR, and MBL are crucial for platelets orchestration of thromboinflammation in stroke (**Figure 1**). Therefore, corresponding translational approaches that may provide novel therapeutic strategies in stroke treatment and prevention are depicted further below.

## Contribution of Platelets to Neurovascular Inflammation in Neurodegenerative Diseases

Neuroinflammation has been associated with a variety of further diseases including amyotrophic lateral sclerosis (ALS), epilepsy, traumatic brain injury, Parkinson’s disease, and Huntington’s chorea (149) but also with non-neurological chronic conditions such as rheumatoid arthritis, obesity and diabetes (150, 151). While the contribution of platelets to central nervous system (CNS)-inflammation in some of these diseases has recently been reviewed elsewhere (152), this review focuses on MS and Alzheimer’s disease (AD).

### Platelets in Experimental Autoimmune Encephalomyelitis and Multiple Sclerosis

MS is a chronic demyelinating and neurodegenerative disease. Although, the pathogenesis of MS is still not completely understood, it is commonly accepted as a heterogeneous, immune-mediated condition which is caused by gene–environment interactions (153). Focal areas of demyelination (plaques) constitute a pathological hallmark of MS. These areas are typically characterized by breakdown of the blood-brain barrier (BBB), whereby antigen­presenting cells (APCs) such as B cells and myeloid cells (macrophages, dendritic cells and microglia) pass through the BBB and initiate the differentiation of memory T cells into pro-inflammatory T helper (Th) lymphocytes (Th1 and Th17). Subsequent recruitment of inflammatory effector cells into the CNS parenchyma is mediated by leukocyte or endothelial adhesion molecules and accompanied by pro-inflammatory stimulation of microglial cells which promotes destruction of axonal myelin sheath (153).

Platelet abnormalities in MS patients were already reported decades ago (154, 155). These observations are supported by more recent reports that have detected platelet specific GPIIb (CD41) in MS plaque of patients as well as in brain tissue of mice with experimental induced autoimmune encephalomyelitis (EAE, a counterpart of MS in mice) (14, 156). Accordingly, cerebrospinal fluid (CSF) levels of PAF have been correlated with both EAE (157) and MS disease activity (158). Interestingly, PAF receptor knockout have yielded a diminished severity of inflammation and demyelination in EAE mice (157). Recently, it was demonstrated that brain-abundant gangliosides GT1b and GQ1b were specifically recognized by platelets and platelets recognize brain-specific glycolipids in area of perivascular space thereby, triggering immune response cascades (159).

**Figure 2:**
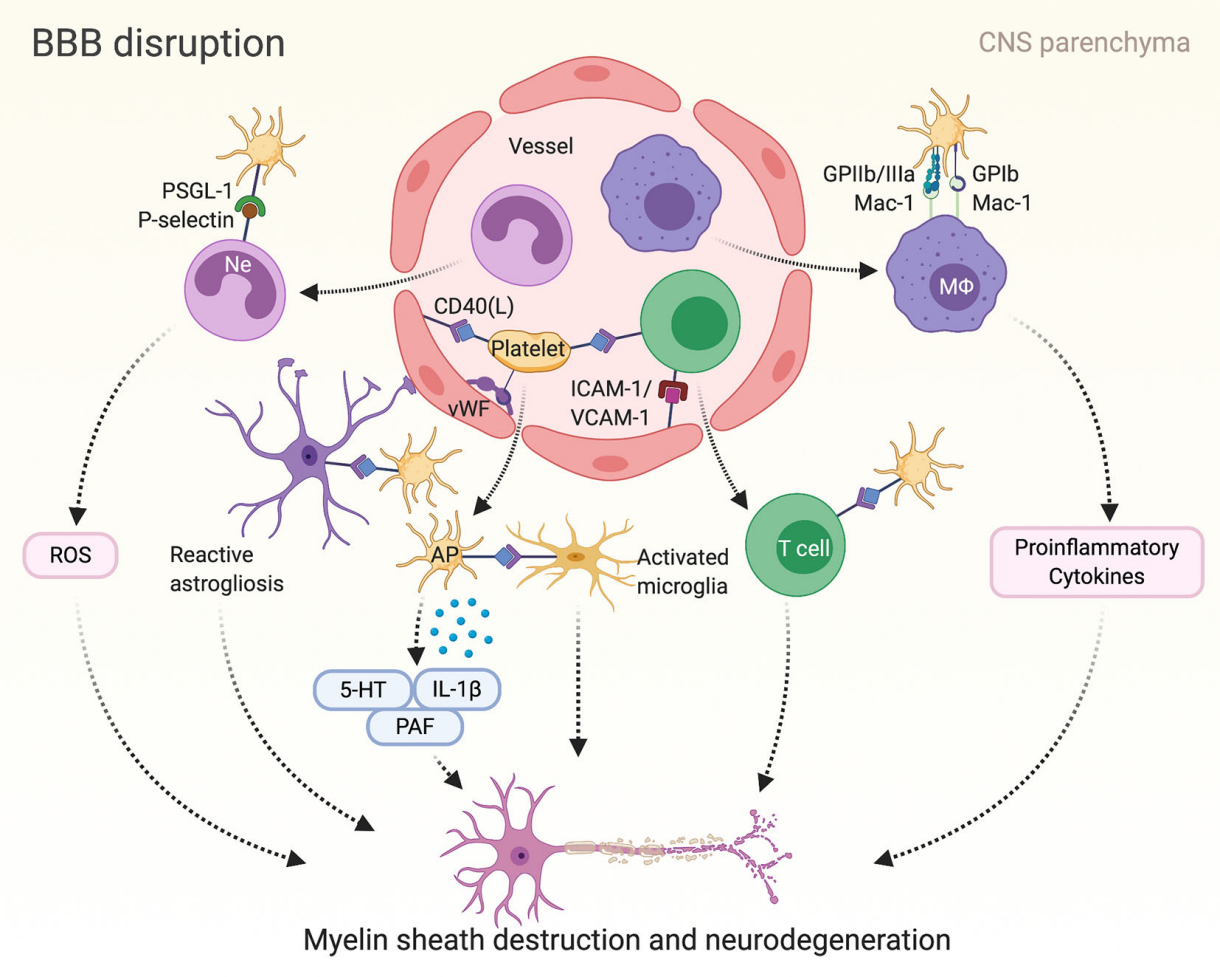
Platelet mediated inflammation in multiple sclerosis (MS) and corresponding mice model of experimental autoimmune encephalomyelitis (EAE): Autoimmune T cells induce the breakdown of the blood-brain barrier (BBB) in multiple sclerosis. Consequently, inflammatory cells such as lymphocytes, macrophages (MΦ) and neutrophils (Ne) penetrate the BBB, promoting reactive activation of astrocytes and microglial cells and finally leading to myelin sheath destruction and axonal damage. Platelets can mediate neuroinflammation in MS/EAE by adhering to the endothelium and interacting with inflammatory and endothelial cells in various ways as depicted here. Furthermore, platelets release serotonin (5-HT), interleukin (IL)-1β and platelet activating factor (PAF), which in turn have been associated with disease progress in MS. AP, activated platelet; GP, glycoprotein; ICAM-1, intercellular adhesion molecule 1; Mac-1, macrophage-1 antigen; PSGL-1, P-selectin glycoprotein ligand-1; ROS, reactive oxygen species; VCAM-1, vascular cell adhesion protein 1; vWF, von Willebrand factor.

Unequivocally demonstrating a crucial contribution of platelets to EAE disease pathogenesis, platelet depletion has attenuated EAE in mice, particularly in the effector phase of the disease; thereby, reducing CNS mRNA levels of CCL-2, CCL-5, CCL-19, CXCR-4, and IL-1β as well as the expression of adhesion molecule ICAM-1 (14) (**Figure 2**). Consistently, recruitment of leukocytes to the inflamed CNS has been diminished by platelet depletion (14, 160). Furthermore, administration of blocking antibodies against GPIIb/IIIa as well as platelet GPIb and its interaction with leukocyte counterreceptor Mac-1 has ameliorated EAE; thus, the involvement of platelets in EAE is regarded to be multi-faceted (14). By contrast, P-selectin is not required for the development of EAE (161).

Besides, serotonin from platelets dense granules may also induce neuroinflammation in EAE, since platelet serotonin has been reported to promote neutrophil recruitment to sites of acute CNS inflammation in mice (162). Remarkably in this context, serotonin transporter depleted mice were less susceptible to EAE (163), and in addition, treatment with selective serotonin-reuptake inhibitor fluoxetine reduced disease activity of relapsing MS patients (164). Interestingly, the secretion of serotonin by platelets has been demonstrated to stimulate differentiation of T cells toward pathogenic Th1, Th17, and interferon-γ/interleukin-17–producing CD4 T cells in a stage-depended manner: Early in MS and EAE, high levels of platelet-derived serotonin stimulate differentiation of pathogenic T cell subsets, promoting proinflammatory responses (165). At the later stages of MS and experimental autoimmune encephalitis, platelets became exhausted in their ability to produce proinflammatory factors and stimulate CD4 T cells but increase their ability to form aggregates with CD4 T cells, thereby decreasing T-cell activation and downmodulating EAE (165).

Furthermore, Sotnikov et al. demonstrated a new role of platelets in in the pathogenesis of EAE as P-selectin on platelets can interact with sialated glycosphingolipids (gangliosides) that are integrated in astroglial and neuronal lipid rafts which may constitute a new type of the neuronal damage danger signal (159). During neuroinflammation, platelets recognize these specific cerebral glycolipid structures and accumulate in the central nervous system parenchyma triggering further immune response cascades. Fascinatingly, preventing the interaction between platelets and brain-derived lipid rafts in the CNS substantially ameliorated EAE development (159).

Addressing neuropsychiatric symptoms of MS, such as anxiety and depression, it has recently been shown that GPIb antibody-mediated platelet depletion prevented the EAE-induced increase in anxiety-like behavior which was associated with reduction of the pro-inflammatory environment to control levels in the hippocampus of mice (166).

However, it is suggested that platelets are only one player in the interaction of coagulatory and thrombo-inflammatory systems with neuroinflammation in MS. For instance, tissue factor as well as thrombin were highly expressed in chronic active lesions of MS patients (167). Interestingly, thrombin inhibition by hirudin has ameliorated EAE (167). Furthermore, Göbel et al. have reported that deposition of FXII is detectable in CNS tissue of MS patients (168). Grippingly, deficiency, or pharmacologic blockade of FXII have rendered mice less susceptible to EAE (168). Considering the above depicted interaction of platelets and FXII, a FXII-mediated contribution of platelets to EAE might be feasible.

To recapitulate, both platelet GPIIb/IIIa and GPIb receptor embody promising targets for future MS therapy. Furthermore, the P2Y_12_ receptor antagonists clopidogrel and ticagrelor have recently been shown to alleviate disease severity of EAE in mice (169). However, neither glycoprotein inhibitors nor ADP receptor antagonists have yet been investigated in clinical trials for treatment of MS patients. But interestingly, glatiramer acetate (Copaxone), an FDA and EMA approved drug for the treatment of MS, has been demonstrated to inhibit thrombin-induced calcium influx in human and mouse platelets. Furthermore, glatiramer acetate also decreased thrombin-induced PECAM-1, P-selectin, and active form of GPIIb/IIIa surface expression and formation of platelet aggregates for both mouse and human platelets, suggesting that glatiramer acetate inhibit neuroinflammation by affecting not only immune cells but also platelets (170).

### Implications of Platelet Activation for Alzheimer’s Disease

AD is a neurodegenerative brain disorder that slowly leads to severe cognitive impairment. The neuropathological hallmarks of AD constitute the formation of intracellular neurofibrillary tangles and the deposition of amyloid-ß (Aβ) in brain tissue and cerebral vessels (so-called cerebral amyloid angiopathy, CAA), accompanied by neuroinflammation as well as neuronal and synaptic loss. Interestingly, platelets constitute the primary source of amyloid-ß peptide (Aß) and its precursor protein, amyloid precursor protein (APP), in the blood (171, 172), as they are secreted following platelet activation (173, 174). Evidence suggests that both and APP play a role in regulating thrombosis and hemostasis (175, 176).

Two decades ago enhanced platelet activation was demonstrated in AD patients (177). Later, this was referred to an increased lipid peroxidation (178). In accordance, platelets have shown enhanced activity and increased adhesion to subendothelial matrix components in transgenic mice model of AD (179, 180). Further pointing to a pathophysiological relevance of platelets in AD progression, activity of ß-secretase, an enzyme which is required for the cleavage of APP, has been shown to be elevated in peripheral blood platelets of patients suffering AD compared to controls (181).

Interestingly, prior to Aß plaque formation, aggregated platelets were shown as a first pathological sign in AD mouse model, suggesting platelets as therapeutic target in early AD (182). Indeed, Donner et al. found that synthetic monomeric Aβ_40_ can bind through its RHDS (Arg-His-Asp-Ser) sequence to GPIIb/IIIa, stimulating the secretion of ADP and the chaperone protein clusterin from platelets (183). This was accompanied by the formation of fibrillar Aβ aggregates and further Aβ_40_ binding to platelets in a feed-forward loop (183). Strikingly, clopidogrel inhibited Aβ aggregation in platelet cultures; and further, platelet inhibition diminished the amount of clusterin in the circulation as well as the incidence of CAA in transgenic AD model mice (183). Underscoring anti-platelet drugs potential as useful therapeutic targets in counteracting CAA and AD, it has been demonstrated that platelets isolated from AD mice promote severe vessel damage, matrix metalloproteinases activation and neuroinflammation in wildtype mice brain, in an organotypic *ex vivo* brain slice model, thereby inducing Aß-like immunoreactivity at the damaged vessel sites (184).

Beyond the illustrated potential therapeutic relevance of platelets, recent metabolomic analysis revealed that platelet phosphatidylcholines constitute promising biomarkers to diagnose AD (185) and CAA (186).

## Platelets in the Modulation of Neuronal Electric Activity, Synaptic Functions, and Plasticity

As already discussed, brain-enriched glycosphingolipids within neuronal lipid rafts were shown to induce platelet degranulation and secretion pro-inflammatory factors (159). In traumatic brain injury (TBI) - induced inflammation model the interaction of platelets with neuronal lipid rafts has been displayed to stimulate neurite growth, increase the number of postsynaptic Sontikov Idensity protein 95-positive dendritic spines, and intensify neuronal activity (187). Using adoptive transfer and blocking experiments the authors demonstrated that platelet-derived serotonin and platelet activating factor play a key role in regulation of neuroinflammation and neuronal plasticity after TBI (187).

With respect to the modulation of neuronal electric activity, a more recent study demonstrated that platelets substantially enhance epileptic seizures in a mouse model of pentylenetetrazole (PTZ) -induced seizures (188). Thereby, platelets ecreted serotonin, contributed to increased BBB permeability. In addition, platelets directly stimulated neuronal electric activity and induced the expression of genes related to early neuronal response and neuroinflammation. Grippingly, intracranial injection of platelets was sufficient to induce severe seizures, demonstrating to a novel role of platelets in the development of epileptic seizures, and pointing to potential new therapeutic approaches by targeting platelets to prevent and treat epilepsy (188).

## Potential Translational and Clinical Applications

To date, patients with non-cardioembolic ischemic stroke or transient ischemic attack (TIA) receive antiplatelet therapy with acetylsalicylic acid (aspirin) or clopidogrel for secondary prevention (189). However, experimental *in vivo* studies in mice have revealed that treatment with ticagrelor reduces infarct size and recovers neurological function after tMCAO to a greater extent than aspirin (190). Nevertheless, the SOCRATES clinical trial demonstrated that ticagrelor is not superior to aspirin in reducing the rate of stroke, myocardial infarction, or death at 90 days after acute ischemic stroke or TIA (191). However, current results of the THALES trial have demonstrated that the risk of the composite of stroke or death within 30 days in patients with a mild-to-moderate acute noncardioembolic ischemic stroke or TIA was lower with ticagrelor and aspirin than with aspirin alone, while severe bleeding was more frequent with ticagrelor (192).

Emphasizing the portrayed role of GPVI, the novel GPVI-Fc fusion protein Revacept, which blocks the collagen target for GPVI binding, has been shown to improve cerebral infarct volume and functional outcome in murine stroke model (193). Furthermore, Revacept has enhanced the efficacy of thrombolysis treatment after tMCAO in mice (194). Therefore, a clinical phase II trial aims to examine whether patients suffering from symptomatic carotid artery stenosis, TIA or stroke take advantage of Revacept plus antiplatelet therapy compared to antiplatelet therapy alone (NCT01645306). A further phase II trial will assess the efficacy and safety of Revacept in patients undergoing elective PCI (195). In addition, a complete blockade of platelet GPVI using a monoclonal anti-GPVI antibody (ACT017) constitutes an alternative therapeutic approach, although bleeding risk might be higher than in therapy with Revacept (196). Therefore, a clinical phase II trial assessing the safety of ACT017 application in patients with an acute ischemic stroke has recently begun (NCT03803007).

With respect to GPIb, Caplacizumab is an anti-vWF humanized single-variable-domain immunoglobulin (so called nanobody) that inhibits the interaction between ultra large vWF multimers and GPIb on platelets (197). Considering the portrayed significance of the vWF-GPIb axis in preclinical ischemic-reperfusion injury models, caplacizumabs platelet-protective effect in thrombotic thrombocytopenic purpura (TTP) (197) raises hope that this novel vWF-inhibitor might be protective in patients with ischemic stroke as well.

Furthermore, vorapaxar, a PAR-1 inhibitor, has been beneficial in the secondary prevention of atherothrombotic events in a phase III clinical trial (198). However, the PAR-1 inhibitor increased the risk of moderate or severe bleeding, including intracranial hemorrhage; thus, vorapaxar should not be used in persons with history of stroke, transient ischemic attack or intracranial hemorrhage (199). In addition, the PAR-4 inhibitor BMS-986141 is currently being investigated in a phase II trial, examining whether it is effective in reducing the recurrence of stroke in patients that have recently suffered an acute stroke or TIA and receive aspirin (NCT02671461).

Intriguingly, the phosphodiesterase (PDE)-3 inhibitor Cilostazol, which diminishes platelet aggregation by decreasing levels of cyclic adenosine monophosphate (cAMP), has been suggested to reduce stroke recurrence in patients with a prior ischemic stroke (200). In accordance, Bieber et al. have only recently concluded that another novel PDE-3 inhibitor (substance V) protects mice from infarct injury after tMCAO (201). Surprisingly, substance V did not affect platelet function (201).

In respect of MS, the treatment with PDE-4 inhibitor ibudilast (MN-166), that has been reported to inhibit platelet aggregation as well (202), was associated with slower progression of brain atrophy in patients with progressive MS (203). Furthermore, aspirin has latterly been demonstrated to ameliorate EAE in mice (204). As the effect of aspirin on general disease activity is inconclusive (205), further studies are needed to determine the benefits and risks of aspirin but also GPIIb/IIIa, GPIb and P2Y_12_ receptor antagonists in patients with MS.

## Conclusion

In conclusion, growing evidence suggests a crucial involvement of platelets in orchestration of neuroinflammation. Therefore, platelets could be considered as immune cells. A broad range of recent experimental approaches indicate that platelets participate in pathogenesis of AD, MS, and stroke associated neuroinflammation. Expanding our knowledge about these novel concepts will help to further understand mechanisms of neuro-inflammatory diseases and could reveal feasible therapeutic strategies with the aim of improving patient’s quality of life.

## Author Contributions

ER wrote the manuscript in consultation with HL. HL conceptualized and submitted the manuscript. All authors contributed to the article and approved the submitted version.

## Funding

HN is supported by the Clinician Scientist Programme of the DZHK (German Research Centre for Cardiovascular Research), partner site Hamburg/Lübeck/Kiel.

## Conflict of Interest

The authors declare that the research was conducted in the absence of any commercial or financial relationships that could be construed as a potential conflict of interest.

## Abbreviations

5-HT, serotonin; ADAMTS13, a disintegrin and metalloprotease with thrombospondin type 1 repeats 13; ADP, adenosine diphosphate; ALS, amyotrophic lateral sclerosis; AP, activated platelet; ApoE, apolipoprotein E; BBB, blood-brain barrier; BK, bradykinin; C3aR, complement receptor for C3a; C5aR, complement receptor for C5aR; cAMP, cyclic adenosine monophosphate; CCL, CC-chemokine ligand; CD40L, CD40 ligand; CLEC-2, C-type lectin-like receptor-2; CNS, central nervous system; CSF, cerebrospinal fluid; CX3CL1, chemokine (C-X3-C motif) ligand 1, fractalkine; CXCL, chemokine (C-X-C motif) ligand; DC, dendritic cell; EAE, experimental autoimmune encephalomyelitis; F, coagulation factor; FasL, Fas ligand; FasR, Fas receptor; FOXP3, Forkhead box P3; GP, glycoprotein; HIV, human immunodeficiency virus; HUVEC, human umbilical vein endothelial cell; ICAM, intercellular adhesion molecule; IL, interleukin; JAM-C, junctional adhesion molecules-C; KKS, kallikrein–kinin system; LFA-1, lymphocyte function-associated antigen 1; LPS, lipopolysaccharide; Mac-1, macrophage-1 antigen; MBL, mannan-binding lectin; MCP1, monocyte chemotactic protein 1; MRI, magnetic resonance imaging; MS, multiple sclerosis; MΦ, macrophage; Ne, neutrophil; NF-κB, nuclear factor kappa-light-chain-enhancer of activated B cells; PAF, platelet activating factor; PAMP, pathogen-associated molecular pattern; PAR, protease-activated receptor; PCI, percutaneous coronary intervention; PDE, phosphodiesterase; PECAM-1, platelet endothelial adhesion molecule-1; PF4, platelet factor 4; PLA, forming platelet-leukocyte-aggregates; PMP, platelet-derived microparticle; polyP, polyphosphates; PPX, recombinant *Escherichia coli* exopolyphosphatase; PSGL-1, P-selectin glycoprotein ligand-1; RANTES, regulated and normal T cell expressed and secreted; ROS, reactive oxygen species; sCD40L, soluble CD40L; TF, tissue factor; TIA, transient ischemic attack; TJs, tight junctions; TLR, toll-like receptor; tMCAO, transient middle cerebral artery occlusion; TNF, tumor-necrosis factor; Treg, regulatory T cell; TTP, thrombotic thrombocytopenic purpura; VCAM, vascular cell adhesion protein; vWF, von Willebrand factor; WT, wild-type.
